# Surgeons’ Perspectives on Intraoperative Biopsy in Perforated Gastric Ulcers: A Nationwide Survey from Türkiye

**DOI:** 10.3390/healthcare14101323

**Published:** 2026-05-13

**Authors:** Adem Tuncer, Cuneyt Kayaalp, Servet Karagul

**Affiliations:** 1 Department of General Surgery, Florence Nightingale Hospital, Demiroğlu Bilim University, Istanbul 34365, Turkey; ademtuncer89@hotmail.com; 2 Department of General Surgery, Faculty of Medicine, Istanbul Atlas University, Istanbul 34403, Turkey; cuneytkayaalp@hotmail.com

**Keywords:** perforated gastric ulcer, intraoperative biopsy, malignancy risk, postoperative endoscopy

## Abstract

**Objective:** Intraoperative biopsy in perforated gastric ulcers has traditionally been widely used to exclude the risk of malignancy. However, despite accumulating evidence in recent years indicating a low incidence of malignancy, it remains unclear to what extent this approach has been adopted by surgeons. This study aims to evaluate the attitudes of general surgeons in Türkiye toward intraoperative biopsy in perforated gastric ulcers and to identify the factors influencing this decision. **Materials and Methods:** A descriptive, cross-sectional, nationwide survey was conducted among actively practicing general surgeons in Türkiye. A total of 361 surgeons were included. The structured questionnaire addressed demographic data, biopsy practices in perforated gastric ulcers, preferred surgical approaches, and postoperative endoscopy planning. Multivariable logistic regression analysis was performed to determine factors associated with routine intraoperative biopsy. **Results:** The mean age of participants was 41.4 ± 10.3 years, and the mean duration of surgical experience was 12.8 ± 9.7 years. Only 34.07% of surgeons correctly estimated the current malignancy risk (<5%) in perforated gastric ulcers; 115 participants (28.3%) did not provide a quantitative estimate. Among respondents, 54.57% reported performing routine intraoperative biopsy, 41.55% reported performing selective biopsy, and 3.88% reported performing no biopsy. Surgeons performing routine biopsy were older (42.6 ± 10.1 vs. 39.8 ± 10.1 years, *p* = 0.01) and more experienced (14.3 ± 9.7 vs. 11.0 ± 9.2 years, *p* = 0.002). In multivariable logistic regression, age (OR 1.02, 95% CI 0.95–1.10, *p* = 0.608), surgical experience (OR 1.01, 95% CI 0.93–1.09, *p* = 0.820), and perceived malignancy risk (OR 1.02, 95% CI 0.99–1.05, *p* = 0.116) were not independent predictors of routine biopsy. Postoperative endoscopy was recommended by 87.53% of participants. **Conclusions:** Routine intraoperative biopsy for perforated gastric ulcers remains widely practiced in Türkiye despite the low malignancy rates reported in the contemporary literature. Surgeons’ biopsy decisions are largely influenced by perceived malignancy risk, experience, and traditional clinical approaches. These findings suggest that an evidence-based strategy relying on selective biopsy and planned postoperative endoscopy, rather than routine intraoperative biopsy, should be more effectively integrated into clinical practice.

## 1. Introduction

The global prevalence of peptic ulcer disease is approximately 5–10%, and gastric ulcers occur less frequently than duodenal ulcers [1]. Perforation develops in approximately 2–10% of all peptic ulcer cases and represents a serious complication requiring emergency surgery [2,3,4]. While the risk of an underlying duodenal malignancy in duodenal ulcer perforation is negligible, it has long been recognized that gastric ulcers may be associated with gastric cancer. Therefore, routine intraoperative biopsy during gastric ulcer perforation has traditionally been considered necessary to avoid missing malignancy. This approach is largely based on older studies reporting gastric cancer rates of 9% to 23% in patients with perforated gastric ulcers [5,6].

Today, this traditional approach continues to be widely used due to established clinical habits. However, accumulating evidence over the past decade has begun to question this practice. Recent studies indicate that the incidence of malignancy in perforated gastric ulcers is much lower than previously thought and is generally below 5% [1,2,7]. Furthermore, obtaining an intraoperative biopsy from the perforation site during emergency surgery may increase operative time, contamination risk, and morbidity [8]. Intraoperative biopsy may contain poor diagnostic material, resulting in diagnostic and decision-making difficulties. Finally, the growing recognition of the importance of neoadjuvant therapy in gastric cancer surgery has shifted practice away from prioritizing radical surgery [9,10]. These findings suggest that the traditional approach of routine intraoperative biopsy may no longer be necessary in every case and should be re-evaluated in light of modern data. There are no local guidelines in Türkiye regarding intraoperative biopsy in gastric ulcer perforation. Here, we have created the possibility of conducting a nationwide survey for the first time. Furthermore, although there is no clear recommendation in the literature for intraoperative biopsy in gastric perforation, there is a trend toward endoscopic biopsy after healing [7].

Despite increasing evidence supporting a more selective approach, there is a lack of data on how these findings are reflected in real-world surgical practice. In particular, the extent to which surgeons’ perception of malignancy risk influences intraoperative decision-making remains unclear. Understanding this gap between evidence and practice is essential for improving clinical protocols and patient outcomes. To assess current clinical attitudes and practices and to determine whether outdated beliefs regarding malignancy risk persist, we conducted a nationwide survey among general surgeons in Türkiye. Our aim was to evaluate how frequently routine biopsy is performed during emergency surgery for perforated gastric ulcers and to investigate the factors influencing this decision. We also sought to raise awareness of recent evidence and contribute to improving surgical protocols in this field.

## 2. Materials and Methods

This study was designed as a descriptive, cross-sectional survey to evaluate current practices and opinions of general surgeons in Türkiye regarding routine intraoperative biopsy during emergency surgery for perforated gastric ulcers. A structured 10-question survey was developed by the research team based on a review of the relevant literature and current guidelines. The full version of the questionnaire is provided in the Supplementary Materials. The survey consisted of three main sections:

(i) Demographic data: Age, years of surgical experience, and city of practice.
(ii) Clinical practices: Whether biopsy is performed in perforated gastric ulcers, preferred surgical techniques (e.g., omentopexy, definitive surgery), and postoperative endoscopy requests.
(iii) Opinions and awareness: Knowledge of recent literature, perception of malignancy risk in perforated ulcers, and factors influencing the decision to perform biopsy.

This study received approval from the İstanbul Aydın University Non-Interventional Clinical Research Ethics Committee on 20.06.2025 (approval no: 136/2025). All procedures were conducted in accordance with the Declaration of Helsinki. Participants were informed prior to the survey, and participation was voluntary.

**Figure 1:**
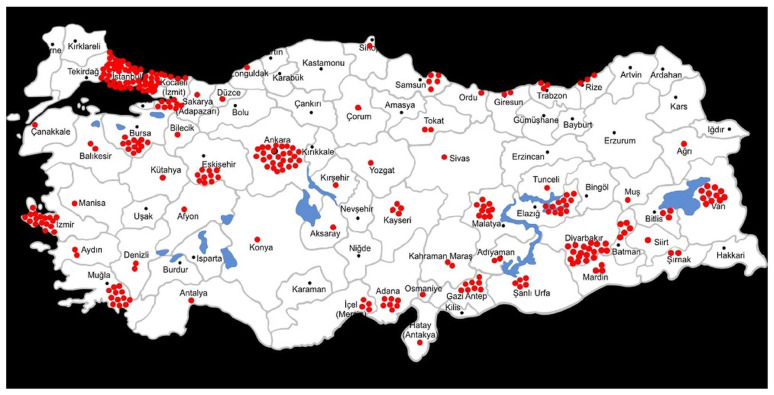
Geographic distribution of general surgeons participating in the survey across Türkiye (each dot represents one general surgeon).

The study population consisted of general surgeons actively working in various institutions across Türkiye (university, training and research, state, and private hospitals). The geographical distribution of participants was visualized such that each point represented one surgeon, with density reflecting participant numbers (Figure 1). Assuming approximately 5000 active general surgeons in Türkiye, the required sample size was calculated using G-Power 3.1 software at a 95% confidence level and 5% margin of error, targeting a minimum of 357 participants.

The survey was pilot-tested on a small group of surgeons (*n* = 11) to assess clarity and appropriateness, followed by minor revisions based on feedback. Following pilot testing (*n* = 11), minor wording revisions were carried out to improve clarity. Formal reliability testing (e.g., Cronbach’s alpha) was not performed, which represents a limitation. These 11 cases were excluded from the main analysis. Perceived malignancy risk was assessed using an open-ended numerical question. Biopsy practice was categorized as routine, selective, or none based on predefined response options. Selective biopsy was defined as performing a biopsy based on intraoperative findings, such as suspicious macroscopic appearance or ulcer location. The survey was administered online via a secure platform (Google Forms) and distributed during general surgery meetings. The survey link was shared through professional surgical associations (Turkish Surgical Society), academic networks, and direct email invitations. Participation was voluntary, and all responses were anonymous. Inclusion criteria were limited to actively practicing general surgeons in Türkiye. Participants who did not provide a quantitative estimate of malignancy risk (*n* = 115) were excluded from analyses involving continuous risk estimation, but they were included in categorical analyses where applicable. No imputation was performed. Duplicate entries were screened and eliminated. Data collected in person and online were merged into a single Excel dataset. For interval-based responses, arithmetic means were calculated using each participant’s lower and upper values and included in classification analyses.

### Statistical Analysis

Survey data were analyzed using IBM SPSS Statistics, version 25.0 (IBM Corp., Armonk, NY, USA). Descriptive statistics were used to summarize participant characteristics and response distributions. Categorical variables were presented as frequencies and percentages. Chi-square or Fisher’s exact tests were used to evaluate associations between demographic variables and biopsy practices. Continuous variables were compared using Student’s *t*-test or the Mann–Whitney U test according to data distribution. A *p*-value of <0.05 was considered statistically significant.

Multivariable logistic regression analysis was performed to identify independent factors associated with routine intraoperative biopsy. Age, duration of surgical experience, and perceived malignancy risk were included in the model. The results were presented as odds ratios (ORs) with 95% confidence intervals (CIs).

Missing data were handled by complete-case analysis. Participants who did not provide a numerical estimate of perceived malignancy risk (*n* = 115) were retained in all descriptive analyses as a distinct category (“no estimate provided”), but they were excluded from analyses that required a numerical value for this variable, including multivariable regression.

## 3. Results

A total of 361 general surgeons participated in this study. The mean age was 41.4 ± 10.3 years (24–71), and the mean surgical experience was 12.8 ± 9.7 years (0.3–45 years). Surgeons estimated the potential malignancy risk in gastric ulcer perforation as 9.1 ± 10.9% (0–80%). While 34.07% correctly estimated the expected malignancy rate of <5%, the majority either overestimated or did not express an opinion (*n* = 115). There was no difference in age or experience between surgeons who correctly estimated the risk and those who did not.

Among participants, 54.57% (*n* = 197) reported routinely performing biopsy during perforation repair, 41.55% (*n* = 150) performed selective biopsy, and only 3.88% (*n* = 14) did not perform biopsy. The two main factors influencing the decision to biopsy were the appearance of the perforation (85.3%) and its location (85.3%), whereas defect size (27.7%) and patient age (16.9%) were less influential (Table 1).

Surgeons performing routine biopsy were older and had more surgical experience (42.6 ± 10.1 years and 14.3 ± 9.7 years) compared with those who did not (39.8 ± 10.1 years and 11.0 ± 9.2 years) (*p* = 0.01 and *p* = 0.002, respectively). However, in multivariable analysis, these factors were not independently associated with routine biopsy. There was also a linear relationship between predicted malignancy risk and biopsy practices (Table 2). Most participants (*n* = 328) preferred primary repair (e.g., omental patch). Twenty-one recommended resection, and 12 responded “depends on the case.” Surgeons recommending resection were older and more experienced (49.1 ± 10.6 years and 20.9 ± 9.9 years) compared with those preferring primary repair (40.8 ± 10.1 years and 12.2 ± 9.5 years) (*p* < 0.002 and *p* = 0.001, respectively) (Table 2).

In multivariable logistic regression analysis, age, surgical experience, and perceived malignancy risk were associated with routine intraoperative biopsy; however, these associations did not reach independent statistical significance (Table 3). The direction of the point estimates was consistent with the univariate findings, but the confidence intervals were wide and crossed unity, precluding the identification of a single dominant independent predictor. This should be interpreted with caution: the univariate associations with age and experience remain clinically relevant, but our data do not permit the conclusion that any of these variables independently drives biopsy behaviour once the others are accounted for.

The vast majority of participants (87.53%, *n* = 316) recommended endoscopy after perforation. The primary reasons were evaluation of malignancy risk (84.5%), assessment of reflux or hiatal hernia (35.7%), and general follow-up (35.7%). Endoscopy for Helicobacter pylori investigation was recommended by 21.6%.

Consistent trends were observed between biopsy practices and postoperative endoscopy planning. Of 197 participants who reported performing biopsies in every patient, 177 (89.8%) recommended postoperative endoscopy for all patients. Of 150 participants performing biopsy only in suspicious cases, 125 (83.3%) still recommended endoscopy for all patients. All participants (100%) who reported never performing a biopsy planned postoperative endoscopy for every patient (Table 4).

No participant who avoided biopsy failed to recommend postoperative endoscopy. These findings demonstrate a strong parallel between ulcer biopsy practice and postoperative endoscopy planning.

Surgeons’ estimates of patient compliance with postoperative endoscopy varied: 41 expected 0–25% compliance, 79 expected 26–50%, 119 expected 51–75%, and 119 anticipated 76–100% compliance.

## 4. Discussion

In this study, the attitudes and behaviors of general surgeons in Türkiye regarding intraoperative biopsy in perforated gastric ulcers were evaluated. Contemporary data indicate that the detection rate of malignancy through intraoperative biopsy in acute gastric perforations is quite low and that the clinical contribution of routine biopsy may be limited [11]. In a long-term cohort analysis by Koca et al., excisional biopsy performed for perforated gastric ulcers was associated with a significant increase in postoperative complications, while intraoperative biopsies did not contribute to malignancy diagnosis [12]. According to Amoli et al., the rate of malignancy detection in intraoperative biopsies taken from perforated prepyloric gastric ulcers was extremely low, and the authors emphasized that the diagnostic contribution of routine biopsy may be limited [13]. The World Society of Emergency Surgery’s (WSES) guidelines also emphasize that exclusion of malignancy in hemodynamically stabilized patients should be performed not during emergency surgery but through planned postoperative upper gastrointestinal endoscopy and targeted biopsies. Concerns have also been raised about the theoretical risk of tumour cell dissemination from biopsy of a perforated malignancy; although the clinical magnitude of this risk is uncertain, it has contributed to guideline recommendations to defer tissue diagnosis until formal postoperative endoscopy [14]. In light of these findings, a strategy based on selective biopsy in the presence of macroscopic suspicion and systematically planned early postoperative endoscopy in all patients appears to be more rational and evidence-based than routine intraoperative biopsy in every case. In a recent retrospective study by Trindade et al., intraoperative biopsies were reported to have limited contribution to malignancy diagnosis in patients operated on for perforated gastric ulcer, whereas postoperative upper gastrointestinal endoscopy played a decisive role in excluding malignancy and establishing a definitive diagnosis [15].

It is noteworthy that a considerable proportion of participants (*n* = 115) did not provide a quantitative estimate of malignancy risk. This may not necessarily indicate a lack of knowledge but rather reflect uncertainty and clinical concern in the emergency surgical setting. The wide variability of malignancy rates reported in the literature and the absence of a definitive threshold value may have led surgeons to avoid specifying a risk percentage. In this context, participants who did not express an opinion may represent clinical uncertainty.

In this study, three-quarters of participants overestimated the current malignancy risk, and approximately 96% reported continuing to perform biopsies either routinely or selectively. This suggests that surgeons’ decisions are still largely shaped by outdated information or intuitive risk perception. Such an approach carries the risk of leading to unnecessary biopsies and, consequently, potential surgical complications. The disadvantages of intraoperative biopsy—including prolonged operative time, increased complication risk, and limited tissue quality due to inflammation—are well recognized [7,8,12]. In a randomized study by Lau JY et al., surgical trauma in patients undergoing repair for perforated peptic ulcer was shown to significantly affect systemic inflammatory response and endotoxemia levels [16]. This finding suggests that additional interventions during emergency surgery may increase physiological burden. Therefore, a strategy aligned with contemporary surgical and oncological principles is to prioritize perforation repair, followed by diagnostic confirmation through endoscopic evaluation, and subsequent treatment planning within a multidisciplinary framework [2,10].

A radical transformation has occurred in gastric cancer treatment, particularly over the last decade. Neoadjuvant (preoperative) chemotherapy has become the standard approach in locally advanced gastric cancer. Randomized controlled trials such as the MAGIC study have demonstrated significant improvements in resectability and survival among patients receiving perioperative therapy. Since the MAGIC trial more than two decades ago, perioperative chemotherapy has been the standard of care for locally advanced resectable gastric cancer [9]. As a result, primary surgery has often been replaced by chemotherapy following endoscopic diagnosis, with elective surgery planned as a second stage. Therefore, in a patient presenting with perforation who is subsequently diagnosed with gastric cancer, performing simple repair rather than resection during the initial operation reduces morbidity and allows the patient to be directed to a more appropriate oncological protocol. In patients undergoing immediate resection, the opportunity for neoadjuvant therapy is lost, and an important prognostic step may be missed. Furthermore, resection may increase postoperative complication risk when severe contamination is present at the perforation site [8]. These realities suggest that making a resection decision based on suspected malignancy identified through intraoperative biopsy may no longer be an oncologically preferable strategy. The current ideal approach is to stabilize the patient through perforation repair, perform a high-quality endoscopic evaluation, and, if malignancy is confirmed, plan treatment (neoadjuvant therapy followed by surgery) according to oncological principles [9,10,17]. Mortality in perforated peptic ulcer is known to be strongly influenced by the patient’s physiological condition and accompanying risk factors. The Peptic Ulcer Perforation (PULP) score described by Møller et al. has been reported as an effective tool for predicting mortality risk in this patient group [18]. This supports the notion that, in emergency surgical management, priority should be given to the patient’s overall risk profile and stabilization rather than oncological suspicion.

The finding that the most influential factors in surgeons’ biopsy decisions were the macroscopic appearance and anatomical location of the perforation indicates that surgeons continue to rely heavily on intraoperative observation and clinical intuition. Conversely, the relatively limited influence of more objective factors, such as defect size and patient age, suggests that biopsy practice is not standardized and is largely based on individual judgment.

Postoperative endoscopy is an effective and safe method for excluding malignancy and evaluating Helicobacter pylori infection, reflux, or other accompanying conditions [19,20,21]. The majority of participants reported recommending postoperative endoscopy, most commonly due to concerns about malignancy (84.5%), indicating that surgeons wish to complete the diagnostic process postoperatively even when an intraoperative biopsy is performed. Recommendations for postoperative endoscopy for non-malignant indications (such as reflux, Helicobacter pylori infection, and general evaluation) suggest that surgeons use endoscopy not solely to exclude malignancy but also as a broader diagnostic tool. However, responses regarding patient compliance revealed that surgeons have notable concerns about the practical feasibility of postoperative endoscopy. The frequent recommendation of postoperative endoscopy despite intraoperative biopsy suggests an attempt to secure the diagnostic process at multiple stages. This approach may be associated with an effort to reduce diagnostic uncertainty and manage clinical responsibility more cautiously. The findings indicate that merely recommending endoscopic follow-up is insufficient; rather, it should be organized as a systematic follow-up process. Whether surgeons who anticipate low postoperative endoscopy compliance rely more heavily on intraoperative biopsy is an important question that warrants further investigation. Nevertheless, approximately one-third of respondents expected that half or fewer of their patients would actually attend postoperative endoscopic follow-up. This is a striking finding: a substantial barrier to adopting an evidence-based strategy of selective biopsy plus planned postoperative endoscopy may not be surgeon preference at all, but rather a legitimate concern about the reliability of the outpatient follow-up system.

This study has several limitations. Economic factors, such as reimbursement policies, were not evaluated in this study and may also influence intraoperative decision-making. The reliance on self-reported data may not fully reflect actual clinical practice. Additionally, because the survey was voluntary, the results may contain sampling bias. Nevertheless, the findings provide important insight into how closely surgical practice in Türkiye aligns with contemporary evidence. Malignancy rates in perforated gastric ulcers may vary across regions due to differences in gastric cancer incidence and screening practices. This variability may partially explain differences in surgeons’ perception of malignancy risk. Additionally, the voluntary nature of participation introduces potential selection bias, possibly favoring academically active surgeons. This study reflects self-reported practices rather than actual clinical behavior, which may introduce recall bias and social desirability bias. Furthermore, factors such as hospital type, availability of endoscopy, and institutional protocols were not assessed and may influence decision-making.

This study also found that older and more experienced surgeons were more inclined toward biopsy and resection. Although univariate analyses showed that these surgeons were more likely to perform a biopsy, these relationships did not reach independent statistical significance in multivariable analysis. This suggests that the biopsy decision is influenced not only by clinical experience but also by entrenched risk perception and traditional knowledge. This indicates that traditional beliefs continue to play a decisive role in clinical decision-making and that integration of new evidence into daily practice may not occur rapidly enough. Surgeons should be informed through updated guidelines, and surgical decisions should be shaped according to evidence-based medicine principles. Similarly, the significantly greater tendency of surgeons who estimated a higher risk of malignancy to perform a biopsy suggests that this decision is primarily driven by perceived risk.

## 5. Conclusions and Recommendations

This study demonstrates that intraoperative biopsy practices for perforated gastric ulcers in Türkiye are not fully aligned with the current literature and contemporary oncological approaches. A notable finding is the discrepancy between surgeons’ reliance on intraoperative biopsy and their limited confidence in postoperative endoscopy compliance. This suggests that the persistence of routine biopsy may reflect not only outdated knowledge but also systemic concerns regarding follow-up reliability. Importantly, the intraoperative diagnosis of malignancy does not mandate immediate resection. Even when malignancy is suspected or confirmed intraoperatively, current oncological principles favor staged management, allowing patients to receive neoadjuvant therapy before definitive surgery.

Accordingly, the primary approach in patients presenting with perforation should be safe repair of the perforation, followed by planned endoscopic and histopathological evaluation to establish a definitive diagnosis and determine the appropriate oncological treatment strategy. Integration of these contemporary approaches into clinical practice is critical for patient prognosis and treatment effectiveness.

Future studies should prospectively evaluate the earliest postoperative time at which upper gastrointestinal endoscopy can be safely and diagnostically performed after a perforated gastric ulcer. Additionally, more comprehensive and multidimensional surveys are needed to better define the clinical, experiential, and perceptual factors influencing surgeons’ decisions regarding intraoperative biopsy and postoperative endoscopy.

Such studies may contribute to standardizing endoscopic follow-up strategies and restructuring perforated gastric ulcer management based on evidence-based principles. These findings highlight the need for targeted educational interventions and the development of standardized national guidelines to bridge the gap between evidence and clinical practice.

## Figures and Tables

**Table 1:** Surgeons’ biopsy practices, influencing factors, and indications for postoperative endoscopy.

Question/Category	Yes	Sometimes	No
**Do you perform a biopsy?**	197 (54.57%)	150 (41.55%)	14 (3.88%)
**What influences your decision to perform a biopsy?**			
- Patient’s age	61 (16.9%)	-	300 (83.1%)
- Defect size	100 (27.7%)	-	261 (72.3%)
- Location of perforation	308 (85.3%)	-	53 (14.7%)
- Macroscopic appearance of perforation	308 (85.3%)	-	53 (14.7%)
**Do you recommend postoperative endoscopy?**	316 (87.53%)	38 (10.53%)	7 (1.94%)
**Why do you recommend postoperative endoscopy?**			
- To evaluate malignancy risk (any)	305 (84.5%)	-	56 (15.5%)
Malignancy alone	130 (36.0%)	-	-
Malignancy + other indications	175 (48.5%)	-	-
- To investigate *Helicobacter pylori*	78 (21.6%)	-	283 (78.4%)
- To assess reflux/gastritis	129 (35.7%)	-	232 (64.3%)
- For general follow-up	129 (35.7%)	-	232 (64.3%)

**Table 2:** Surgeon characteristics associated with biopsy practice, surgical approach, and postoperative endoscopy.

Variable	Group	Value	*p*-Value
Intraoperative biopsy	Routine biopsy	Age: 42.6 ± 10.1	0.01
	Non-routine biopsy *	Age: 39.8 ± 10.1	
	Routine biopsy	Experience: 14.3 ± 9.7	0.002
	Non-routine biopsy *	Experience: 11.0 ± 9.2	
Surgical approach	Primary repair	328 (90.9%)	-
	Resection	21 (5.8%)	
	Case-dependent	12 (3.3%)	
	Resection	Age: 49.1 ± 10.6	<0.002
	Primary repair	Age: 40.8 ± 10.1	
Postoperative endoscopy	Recommended	316 (87.53%)	-
	Selective	38 (10.53%)	-
	No	7 (1.94%)	-

* Non-routine biopsy includes selective biopsy and no biopsy. Values are presented as mean ± SD or *n* (%).

**Table 3:** Multivariable logistic regression analysis of factors associated with performing routine intraoperative biopsy.

Variable	OR	95% CI	*p*-Value
Age (years)	1.02	0.95–1.10	0.608
Surgical experience (years)	1.01	0.93–1.09	0.820
Perceived malignancy risk (%)	1.02	0.99–1.05	0.116

OR: odds ratio; CI: confidence interval. Dependent variable: performance of routine intraoperative biopsy (yes/no).

**Table 4:** Relationship between surgeons’ biopsy practices and their preferences for postoperative endoscopy.

	Yes, for Every Patient (Endoscopy)	No, Only in Suspicious Cases (Endoscopy)	No, I Do Not Consider It Necessary (Endoscopy)
Yes, for every patient (Biopsy)	177 (89.8%)	15 (7.7%)	5 (2.0%)
No, only in suspicious cases (Biopsy)	125 (83.3%)	23 (15.3%)	2 (1.3%)
No, I never perform biopsy (Biopsy)	14 (100%)	0 (0.0)	0 (0.0)

## Data Availability

The data presented in this study are available from the corresponding author upon request.

## Supplementary Materials

The following supporting information can be downloaded at: https://www.mdpi.com/article/10.3390/healthcare14101323/s1, Questionnaire on Physicians’ Perspectives on Intraoperative Biopsy in the Setting of Gastric Ulcer Perforation.
