# Supplementary material for: Surgeons’ Perspectives on Intraoperative Biopsy in Perforated Gastric Ulcers: A Nationwide Survey from Türkiye
Source: Healthcare (Basel). 2026 May 13;14(10):1323. doi: 10.3390/healthcare14101323 (PMC13205359; doi:10.3390/healthcare14101323)
Supplement: Supplementary file 1 [file healthcare-14-01323-s001.zip › healthcare-4205768-supplementary.pdf]

Questionnaire on Physicians’ Perspectives on Intraoperative Biopsy in the Setting of Gastric Ulcer Perforation

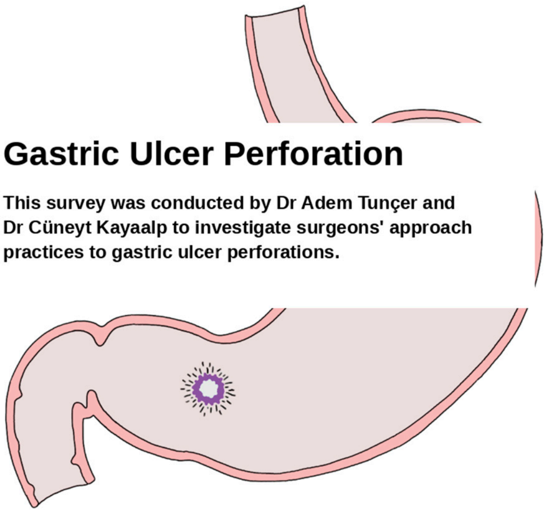

Age: ____________________

City of practice: ____________________

Surgical experience (years): ____________________

1. In your opinion, what is the malignancy rate (%) in perforated gastric ulcers?

   ☐ ____ %

   ☐ No estimate

2. When you encounter a perforated gastric ulcer, do you take a biopsy from the ulcer during surgery?

   ☐ Yes — in every case (routine biopsy)

   ☐ No — only in suspicious cases (selective biopsy)

   ☐ No — I never perform a biopsy

3. In your opinion, which factor most strongly raises the suspicion of malignancy in a perforated gastric ulcer?

   ☐ Patient age

   ☐ Defect size

   ☐ Location of perforation

   ☐ Macroscopic appearance of perforation (induration, irregularity)

   ☐ Other: ____________________

4. What surgical treatment do you perform for a perforated gastric ulcer?

☐ Primary repair (with or without omentopexy)

☐ Ulcer resection

☐ Vagotomy with drainage

☐ Gastric resection

☐ Other: ____________________

5. Do you recommend postoperative endoscopy?

☐ Yes — for every patient

☐ No — only in suspicious cases

☐ No — I do not consider it necessary

6. In your opinion, what is the level of patient compliance with postoperative endoscopy?

☐ 0–25%

☐ 26–50%

☐ 51–75%

☐ 76–100%

7. Please indicate the reasons influencing your decision to perform postoperative endoscopy (you may select more than one):

☐ To evaluate malignancy risk (exclusion of malignancy)

☐ To investigate Helicobacter pylori

☐ To assess reflux/gastritis

☐ For general follow-up

☐ I do not perform it
